# Gut microbiota and serum metabolic profiles in patients with sepsis-induced cardiomyopathy and their association with the disease

**DOI:** 10.3389/fcimb.2026.1811654

**Published:** 2026-07-10

**Authors:** Kai Chen, Jia Lin, Yizhuo Zhang, Yafang Liu, Xiaojun Yang, Xiaojuan Yang

**Affiliations:** 1 First Clinical Medical College, Ningxia Medical University, Yinchuan, China; 2 Department of Critical Care Medicine, General Hospital of Ningxia Medical University, Yinchuan, China

**Keywords:** gut microbiota, intensive care unit, metabolomics, sepsis, sepsis-induced cardiomyopathy

## Abstract

**Background:**

The pathogenesis of sepsis-induced cardiomyopathy (SIC) remains unclear, and the lack of effective treatment options results in high mortality rates. There may be interactions between gut microbiota dysbiosis, serum metabolic changes, and SIC; however, research in this area is limited. This study aims to provide a theoretical basis for targeted interventions to improve the gut microbiome, thereby enhancing cardiac function and prognosis in patients with SIC.

**Methods:**

This prospective cohort study enrolled 48 septic patients admitted to the intensive care unit (ICU) of a tertiary general hospital in Ningxia, China, between March 2024 and March 2025. Patients were stratified into SIC (n=28) and SEPSIS (n=20) groups. Age- and sex-matched healthy controls (HC, n=10) were also recruited. Fecal samples from all three groups were analyzed via 16S rRNA gene amplicon sequencing, and serum samples from the SIC and SEPSIS groups were analyzed via LC–MS-based untargeted metabolomics. Spearman’s correlations linked microbial and metabolic alterations to echocardiographic parameters and myocardial injury markers.

**Results:**

Compared with the SEPSIS and HC groups, the SIC group presented significant differences in both the α and β diversity of the gut microbiota (all P<0.05). Compared with that in the SEPSIS group, the *Bacillota/Bacteroidota* ratio was significantly lower in the SIC group (P<0.05); compared with the SEPSIS and HC groups, the SIC group presented significantly increased abundances of genera such as *Parabacteroides* and *Clostridium innocuum* group and significantly decreased abundances of genera such as *Oscillospiraceae_UCG-003* and *Lachnospiraceae_UCG-004* (all P<0.05). Forty-nine differential metabolites were identified (VIP≥1, P<0.05): hippuric acid, estrone glucuronide, and TMAO were elevated, while 15-deoxy-Δ12,14-prostaglandin J2 was reduced in the SIC group (all P<0.05). Differential genera/metabolites correlated strongly with SIC diagnostic indicators: e.g., *Parabacteroides* positively correlated with estrone glucuronide, hippuric acid, and GLS, and negatively with LVEF.

**Conclusion:**

Gut microbiota dysbiosis differs between patients with Sepsis-induced cardiomyopathy and healthy individuals or patients with sepsis. Additionally, the serum metabolic profile changes in SIC patients differ from those in patients with sepsis, and these characteristic changes are significantly associated with clinical indicators related to the diagnosis of SIC.

## Introduction

Sepsis is a life-threatening organ dysfunction caused by a dysregulated host immune response to infection (Singer et al., 2016), and the heart is one of the most vulnerable target organs involved in sepsis (Aissaoui et al., 2025). Sepsis-induced cardiac dysfunction is defined as sepsis-induced cardiomyopathy (SIC), which is characterized by diffuse and reversible myocardial dysfunction. SIC complicates 10%–70% of sepsis patients, with a mortality rate of up to 70%–90%, severely impairing the long-term prognosis of septic patients (Khalid et al., 2022; Sato et al., 2025). The underlying mechanism of SIC is complex and not fully understood; the absence of specific targeted therapies hinders prognostic improvement (Sun et al., 2025), making SIC a research hotspot in critical care medicine.

As the largest reservoir of intestinal bacteria and endotoxins in the human body, the gut is recognized as the “engine” of sepsis-associated multiple organ dysfunction syndrome (MODS) (Adelman et al., 2020). Accumulating evidence has demonstrated that gut microbiota dysbiosis plays a pivotal role in the pathogenesis of sepsis-related MODS (Miller et al., 2021; Kain et al., 2024). Whether SIC is accompanied by characteristic gut flora dysbiosis remains to be clarified. Previous studies have confirmed that the structure and composition of the gut microbiota differ significantly between SIC patients and non-SIC sepsis patients (Chen et al., 2022). In addition to bacterial flora, intestinal viral and fungal communities also undergo structural remodeling in SIC patients (Deng et al., 2023). Previous studies have verified the interactions among sepsis, sepsis-related MODS and gut microbiota dysbiosis. Given that the heart is a major target organ of sepsis injury, the unique gut microbial characteristics of SIC patients (compared with those of healthy individuals and simple sepsis patients) may reveal the dynamic correlation between disease progression (from healthy individuals to simple sepsis patients and further to SIC patients) and gut dysbiosis, providing a reference for interventions in the gut microbiota to block disease progression and optimize the clinical prognosis.

Gut microbiota dysbiosis is closely implicated in the pathogenesis of multiple cardiovascular diseases. It participates in the pathological progression of hypertension, atherosclerosis, heart failure and atrial fibrillation by increasing intestinal permeability, promoting lipopolysaccharide (LPS) translocation and triggering chronic systemic inflammation (Trøseid et al., 2020; Witkowski et al., 2020; Paraskevaidis et al., 2023; Luqman et al., 2024). Moreover, gut microbial metabolites exert regulatory effects on cardiac function. Short-chain fatty acids (SCFAs) exert cardioprotective effects through anti-inflammatory activity and intestinal barrier maintenance (De la Cuesta-Zuluaga et al., 2018; Blaak et al., 2020). Elevated circulating TMAO and abnormal bile acid metabolism accelerate the progression of multiple cardiovascular diseases (Vourakis et al., 2021; Spasova et al., 2024). Therefore, exploring the characteristics of gut microbiota dysbiosis, serum metabolic alterations and their disease relevance in SIC can deepen the understanding of SIC pathogenesis and provide a novel strategy for improving SIC prognosis via gut microbiome regulation, which has important clinical translational value.

On the basis of the above background, this study adopted 16S rRNA gene amplicon sequencing to compare the gut microbial composition among SIC patients, non-SIC sepsis patients and healthy controls. Moreover, serum untargeted metabolomics via LC–MS was used to characterize metabolic profile changes in SIC patients. Furthermore, we conducted correlation analyses of differential gut bacterial genera, serum metabolites, echocardiographic parameters and myocardial injury biomarkers to elucidate the intrinsic associations among gut dysbiosis, serum metabolic disorders and myocardial damage in SIC patients.

## Study design and methods

### Study design and participants

This was a prospective observational cohort study recruiting sepsis patients hospitalized in the ICU of the General Hospital of Ningxia Medical University between March 2024 and March 2025.

The inclusion criteria were as follows: ① met the Sepsis 3.0 diagnostic criteria (Singer et al., 2016) and ②were aged ≥18 years. Exclusion criteria: ① Complicated with acute coronary syndrome or receiving cardiac interventional surgery during hospitalization; ② history of chronic heart failure, long-term dialysis-dependent chronic renal insufficiency, or Child–Pugh grade C chronic liver disease; ③ preexisting hypertrophic, dilated or restrictive cardiomyopathy, as well as rheumatic heart disease; ④ history of life-threatening severe arrhythmias (e.g., ventricular tachycardia, ventricular fibrillation); ⑤ unavailability of bedside echocardiography during the ICU; and ⑥ refusal of enrollment or voluntary withdrawal by patients or their legal guardians.

This study was approved by the Ethics Committee of the General Hospital of Ningxia Medical University (Approval No. KYLL-2025-0038) and was registered in the Chinese Clinical Trial Registry (Registration No. CTR2500097664). All participants or their guardians signed written informed consent prior to enrollment.

Sample size calculation was performed via beta diversity PERMANOVA (α=0.05, statistical power=0.8, 1:1 matching design) and the method proposed by Kelly et al. (2015). With an expected effect size R2 = 0.3, the estimated sample size per group was 18–20 cases. Owing to limited clinical case enrollment and strict inclusion/exclusion criteria, the final enrollment met the statistical requirements. No prior sample size calculation was performed for metabolomics analysis because of the lack of reference data in the same population.

The diagnostic criteria for SIC are as follows (L'Heureux et al., 2020; Hollenberg and Singer, 2021; Zakynthinos et al., 2025): ① acute onset with potential functional reversal within 7–10 days; ② global biventricular systolic and/or diastolic dysfunction accompanied by decreased myocardial contractility; ③ left ventricular dilatation; ④ poor hemodynamic response to fluid resuscitation and catecholamine therapy; and ⑤ exclusion of acute coronary syndrome as the primary cause of cardiac dysfunction.

Serial echocardiography was performed dynamically to evaluate changes in cardiac function. Professional sonographers completed echocardiographic examinations on days 1, 3 and 5 (if necessary) after ICU admission. Two senior critical care physicians jointly made the SIC diagnosis on the basis of the patients’ clinical history and dynamic echocardiographic parameters.

The enrolled sepsis patients were divided into an SIC group (n=28) and a non-SIC sepsis group (n=20). The HC group (n=10) consisted of age- and sex-matched healthy volunteers with normal echocardiographic results and no underlying cardiovascular, metabolic or gastrointestinal diseases.

### Data collection

Echocardiographic parameters: A GE Vivid E9 ultrasound system was used for cardiac ultrasound examination. The detected parameters included the following: left ventricular global longitudinal strain (GLS), left ventricular ejection fraction (LVEF), and left ventricular outflow tract time–velocity integral (VTI); diastolic function indicators: mitral early diastolic flow velocity (E), mitral late diastolic flow velocity (A), E/A ratio, mitral annular early diastolic tissue velocity (e’), E/e’ ratio, left atrial volume index (LAVI), and peak tricuspid regurgitation velocity (TRV); and right ventricular systolic function indicators: tricuspid annular plane systolic excursion (TAPSE) and tricuspid systolic flow velocity (S). All ultrasound images were acquired by dedicated cardiac sonographers and independently reviewed by two senior echocardiographers in strict accordance with the American Society of Echocardiography guidelines (Lang et al., 2015).

Clinical baseline data: Demographic characteristics, underlying comorbidities, infection sites, and APACHE II and SOFA scores were recorded. Within 24 hours after sepsis diagnosis, laboratory indicators, including the mean arterial pressure (MAP), white blood cell (WBC) count, lymphocyte percentage (LY%), platelet count (PLT), C-reactive protein (CRP), procalcitonin (PCT), blood lactate (Lac), oxygenation index (OI), serum creatinine (CREA), total bilirubin (TBIL), cardiac troponin I (cTnI) and N-terminal pro-B-type natriuretic peptide (NT-proBNP), were collected.

Fecal sample collection and 16S rRNA gene sequencing: Fecal samples were collected upon ICU admission synchronously with echocardiography. For SIC patients, samples within 24 hours after confirmed SIC diagnosis were used for detection; for simple sepsis patients, the first fecal sample after enrollment was analyzed. Genomic DNA was extracted via an E.Z.N.A.^®^ Stool DNA Kit (Omega Biotek, USA) following the manufacturer’s instructions. The DNA concentration and purity were determined via a QUBIT 3.0 fluorometer (Invitrogen, USA). The V3-V4 hypervariable region of the 16S rRNA gene was amplified with the primers 341F (5’-CCTACGGGNGGCWGCAG-3’) and 805R (5’-GACTACHVGGGTATCTAATCC-3’). PCR amplification was performed on an Easy Cycler 96 PCR instrument (Analytik Jena, Germany). The purified PCR products were mixed equally and sequenced on an Illumina NextSeq 2000 platform.

Serum sample collection and LC–MS untargeted metabolomics: Peripheral venous blood samples were collected synchronously with echocardiography upon ICU admission. Serum was isolated by centrifugation at 3000 rpm for 10 min and stored at -80 °C for subsequent detection. Serum samples were thawed slowly at 4 °C, mixed with precooled methanol:acetonitrile:water mixture (2:2:1), vortexed, subjected to low-temperature ultrasonic extraction for 30 min, and then incubated at -20 °C for 10 min. After centrifugation at 14000 × g for 20 min at 4 °C, the supernatant was collected and vacuum-dried. For mass spectrometry detection, the dried sample was redissolved in 100 μL of acetonitrile-water mixture (1:1), vortexed and centrifuged at 14000 × g for 15 min at 4 °C, and the resulting mixture was injected into the sample for analysis. Chromatographic separation was performed on a Vanquish ultrahigh-performance liquid chromatography system coupled with an Orbitrap Exploris™ 480 mass spectrometer (Thermo Fisher, USA). Electrospray ionization was applied in both positive and negative ion modes.

### Data quality control

16S rRNA sequencing data: Raw paired-end sequencing data were sorted by sample and sequencing batch. Cutadapt was used to trim primer sequences and filter abnormal fragments. FastQC and MultiQC were applied to evaluate sequencing quality and base distribution. The quality-filtered sequences were imported into the QIIME 2 (v2024.2) platform and processed via the DADA2 algorithm, including quality trimming, error correction, denoising, paired-end read assembly and chimera removal. Only sequences with overlapping lengths ≥20 bp were retained to generate amplicon sequence variants (ASVs) and abundance profiles. Species annotation was performed via the SILVA 138.2 database, which is based on the naive Bayes classifier. Low-abundance ASVs (sequence count <5 or detected in only one sample) and off-target sequences (mitochondria, chloroplasts, archaea) were removed. Samples with total sequencing reads <4000 were excluded. The average sequencing depth of all the samples was 38528 reads. All samples were normalized to the minimum sequencing depth of 19654 reads for subsequent alpha/beta diversity analysis.

Serum metabolomics data: Raw mass spectrometry data were converted to mzXML format by ProteoWizard. XCMS software was used for peak alignment, retention time correction and peak area extraction. Ion peaks with missing value rates >50% were removed, and missing values were imputed via the K-nearest neighbor algorithm. The feature peaks with a relative standard deviation (RSD) >50% were filtered out. Quality control (QC) sample total ion chromatogram overlap analysis revealed the high stability of instrument detection. Principal component analysis confirmed good clustering repeatability of the QC samples. When the proportion of ion peaks with an RSD ≤30% exceeded 70%, the data were considered suitable for subsequent analysis. Metabolite annotation was performed by matching the exact mass–charge ratio (mass error ≤5 ppm), characteristic fragment ions and retention time with the HMDB and KEGG databases. Metabolite identification confidence strictly followed the four-level criteria of the Metabolomics Standards Initiative (MSI). Targeted MS/MS verification with standard compounds was conducted for key differentially abundant metabolites (Sumner et al., 2007).

### Bioinformatics and statistical analysis

Bioinformatics analysis: Microbial sequencing data were processed via the DADA2 algorithm to obtain ASV abundance tables. Alpha diversity was calculated to evaluate gut microbial richness and diversity. Principal coordinate analysis (PCoA) based on Bray–Curtis, unweighted and weighted UniFrac distances was used to assess intergroup microbial structural differences. PERMANOVA (Adonis) analysis was used to test the statistical significance of community differences. Intergroup differences in microbial relative abundance were analyzed via the Mann–Whitney U test (two groups) and the Kruskal–Wallis H test (three groups). Linear discriminant analysis effect size (LEfSe) was used to screen for biomarker bacterial genera among the groups.

Serum LC/MS non-targeted metabolomics analysis: For the metabolomics data, PCA and orthogonal partial least squares discriminant analysis (OPLS-DA) were performed after total peak area normalization and UV scaling. OPLS-DA model validation was conducted via 200 bootstrap permutations. Differentially abundant metabolites with VIP>1 and P<0.05 (Student’s t test) were identified. KEGG pathway enrichment analysis was performed via the MetaboAnalyst online platform.

Correlation analysis: Spearman’s correlation analysis was used to analyze the correlation of differential bacterial genera and metabolites with echocardiographic parameters and myocardial injury biomarkers. CCA analysis was performed using the `cca` function in the `vegan` package of R. Key differential metabolite profiles were integrated with clinical and physicochemical indicators, such as those associated with SIC diagnosis, to construct a combined metabolic-clinical-environmental factor matrix; microbial community composition at the genus level served as the species matrix. All environmental variables were standardized using UV normalization (Standard Z-score normalization) to eliminate dimensional differences and numerical weighting biases. This approach revealed covariation patterns between microbial data and host multi-omics indicators from a holistic perspective.

Statistical methods: All the statistical analyses were performed via R (v4.5.1) and SPSS 27.0. The Shapiro–Wilk test was used for normality detection. Normally distributed data are presented as the means ± standard deviations and were compared via independent sample t tests; nonnormally distributed data are presented as medians (interquartile ranges) and were analyzed via nonparametric tests. Categorical variables are expressed as case numbers (percentages) and were compared via the chi-square test or Fisher’s exact test. A two-tailed P<0.05 was considered statistically significant.

## Results

### Patient characteristics

This study included a total of 48 patients with sepsis, comprising the SIC group (n=28, 58%) and the SEPSIS group (n=20, 42%). The mean age of the enrolled patients was 54.87 ± 14.66 years, with 30 (62.5%) being male. There were no statistically significant differences between the two groups in terms of sex, age, body surface area, comorbidities, site of infection, APACHE II score, or SOFA score (all P>0.05) (**Table 1**). The HCs (n=10) had a mean age of 55 ± 4.82 years, were male (n=6, 60%), and were fully matched with the enrolled patients. To avoid the influence of confounding factors, intergroup comparisons were performed for several confounding variables; there were no statistically significant differences between the two groups in BMI, endotracheal intubation, or types of antibiotics used (all P>0.05, **Supplementary Table 8**).

**Table 1:** Comparison of baseline characteristics among patients with sepsis-induced cardiomyopathy, sepsis, and healthy controls.

Variable	HC (n=10)	SEPSIS (n=20)	SIC (n=28)	F/χ^2^/Z/t	*P*
Age (years, $\bar{\text{x}}$ ± s)	55.00 ± 4.82	54.80± 15.70	55.00 ± 14.20	0	0.88^b^
Gender				0.04	0.76^b^
Male n (%)	6 (60%)	12 (60%)	18 (64%)		
Female n (%)	4 (40%)	8 (40%)	10 (36%)		
BMI (kg/m^2^, $\bar{\text{x}}$ ± s)		22.79 ± 2.32	24.44 ± 4.25	3.79	0.09^a^
Body surface area (m^2^, $\bar{\text{x}}$ ± s)	1.60 ± 0.20	1.70 ± 0.20	1.80 ± 0.20	-1.00	0.39^b^
Comorbidities					
Diabetes n (%)		5 (25%)	5 (18%)	0.08	0.72^a^
Hypertension n (%)		6 (30%)	8 (29%)	0.01	0.91 ^a^
COPD n (%)		1 (5.0%)	2 (7.1%)	0.04	0.76 ^a^
Coronary heart disease n (%)		3 (15%)	2 (7.4%)	0.12	0.38 ^a^
Cancer n (%)		1 (5.0%)	5 (18%)	0.19	0.19 ^a^
Source of infection					
Hematogenous infection n (%)		4 (20%)	7 (25%)	0.05	0.68 ^a^
Pulmonary infection n (%)		17 (85%)	20 (71%)	0.15	0.27 ^a^
Intra-abdominal infection n (%)		7 (35%)	15 (53.6%)	0.18	0.20 ^a^
Urinary tract infection n (%)		1 (5.0%)	3 (10.7%)	0.10	0.48 ^a^
Infection in other sites n (%)		7 (35%)	5 (17.9%)	0.19	0.17 ^a^
Infectious pathogens					
Gram-positive bacteria n (%)		12 (60%)	15 (53.6%)	0.06	0.65 ^a^
Gram-negative bacteria n (%)		6 (30%)	14 (50%)	0.20	0.16 ^a^
Disease Severity Scores					
APACHE II Score (points, IQR)		22.00 (16.50,26.50)	23.00 (18.00,26.50)	-0.38	0.69 ^a^
SOFA score (points, $\bar{\text{x}}$ ± s)		12.10 ± 3.40	12.10 ± 2.80	-0.10	0.62 ^a^

SIC, Sepsis-induced cardiomyopathy group; SEPSIS, SEPSIS group; HC, Healthy control group; COPD, Chronic Obstructive Pulmonary Disease; APACHE II Score, Acute Physiology and Chronic Health Evaluation; SOFA Score, Sequential Organ Failure Assessment; ^a^: Comparison between the sepsis-associated cardiomyopathy and sepsis groups; ^b^: Comparison among the sepsis-associated cardiomyopathy, sepsis, and healthy control groups.

Analysis of immune function and inflammation-related indicators in both groups revealed that, except for the absolute total T lymphocyte count, which was significantly greater in the SIC group than in the SEPSIS group, there were no significant differences in the other indicators (all P>0.05). Comparisons of organ function-related indicators, such as creatinine and total bilirubin, between the two groups also revealed no statistically significant differences (all P>0.05, **Table 2**)

**Table 2:** Comparison of laboratory data between patients with sepsis-induced cardiomyopathy and patients with sepsis.

Variable	SEPSIS (n=20)	SIC (n=28)	F/χ^2^/Z/t	*P*
Clinical Variables Related to Infection and Immunity				
White Blood Cells (10^9^/L,IQR)	10.20 (6.10,18.90)	9.00 (6,13.6)	-0.84	0.40
Procalcitonin (ng/ml,IQR)	9.00 (0.80,24.00)	15.00 (3.20,37.00)	0.78	0.43
Interleukin-6 (pg/ml,IQR)	130.50 (55.00,606.00)	686.00 (51.90-2108.00)	0.63	0.52
Total T-lymphocyte percentage (%, $\bar{\text{x}}$ ± s)	61.80 ± 15.80	70.00 ± 11.90	-1.93	0.06
Total B-lymphocyte percentage (%, IQR)	18.10 (13.70,27.20)	14.90 (11.70,18.70)	-1.68	0.09
NK cell percentage (%, IQR)	7.70 (4.60,12.00)	8.31 (4.70,10.60)	0.00	1.00
Absolute lymphocyte count (cells/μL, IQR)	566.80 (326.70,807.81)	804.90 (414.71,1051.80)	1.32	0.18
Absolute total T-lymphocyte count (cell/μL, IQR)	192.70 (134.60,354.20)	441.10 (156.70,691.20)	-2.09	0.03*
Absolute total B-cell count (cells/μL, IQR)	104.40 (69.12,147.00)	100.71 (45.21,160.10]	-0.30	0.76
Absolute NK-cell count (cells/μL, IQR)	39.10 (19.70,88.80)	54.40 (31.71,84.00)	0.92	0.35
Serum Immunoglobulin G (g/L, $\bar{\text{x}}$ ± s)	8.10 ± 2.51	8.50 ± 3.20	-0.52	0.60
Serum Immunoglobulin M (g/L, IQR)	1.43 (0.91,2.42)	1.40 (1.00,1.60)	-0.42	0.66
Serum Immunoglobulin A (g/L, IQR)	1.10 (0.51,1.61)	0.90 (0.60,1.20)	-0.12	0.90
Complement C3 (g/L, IQR)	0.71 (0.61,0.95)	0.60 (0.50,0.80)	-1.44	0.14
Complement C4 (g/L, IQR)	0.20 (0.22,0.32)	0.20 (0.10,0.30)	-1.22	0.22
Clinical variables for organ function				
Creatinine (umol/L, $\bar{\text{x}}$ ± s)	95.91 ± 35.12	120.80 ± 64.60	-1.71	0.09
Total Bilirubin (umol/L, IQR)	23.63 (19.25,40.76)	36.21 (19.42,55.92)	0.88	0.37
Lactate (mmol/L, IQR)	1.80 (1.10,2.50)	2.00 (1.41,2.80)	0.94	0.34
Hemoglobin (g/L, $\bar{\text{x}}$ ± s)	112.82 ± 31.61	107.80 ± 18.60	0.63	0.53
Platelets (10^9^/L, $\bar{\text{x}}$ ± s)	117.41 ± 68.62	120.70 ± 64.00	-0.16	0.86

SIC, Sepsis-induced cardiomyopathy group; SEPSIS, SEPSIS group; *P < 0.05.

In the SIC group, echocardiographic variables such as LVEF, absolute GLS values, E, and TAPSE were significantly lower than those in the SEPSIS group (all P<0.05). Compared with those in the SEPSIS group, the levels of myocardial damage markers such as cTnI were significantly greater (P<0.05), and the level of nt-proBNP in the SEPSIS group tended to increase, suggesting impaired cardiac function in SIC patients (**Table 3**).

**Table 3:** Comparison of echocardiographic parameters and cardiac injury markers between patients with sepsis-induced cardiomyopathy and patients with sepsis.

Variable	SEPSIS (n=20)	SIC (n=28)	F/χ^2^/Z/t	P
Echocardiographic Parameters				
GLS ( $\bar{\text{x}}$ ± s)	-16.68 ± 2.04	-10.03 ± 2.63	0.68	<0.001***
LVEF (%, $\bar{\text{x}}$ ± s)	67.77 ± 4.49	52.60 ± 10.27	10.23	<0.001***
VTI (cm/s, $\bar{\text{x}}$ ± s)	19.24 ± 2.77	13.28 ± 3.51	1.05	<0.001***
SV (mL, $\bar{\text{x}}$ ± s)	59.548 ± 11.12	46.5 0 ± 15.78	2.40	0.003**
E (m/s, $\bar{\text{x}}$ ± s)	0.86 ± 0.24	0.70 ± 0.22	0.24	0.026*
A (m/s, $\bar{\text{x}}$ ± s)	0.80 ± 0.24	0.66 ± 0.16	2.61	0.031*
E/A ( $\bar{\text{x}}$ ± s)	1.10 ± 0.37	1.00 ± 0.49	0.23	0.234
e’-S (cm/s, $\bar{\text{x}}$ ± s)	8.85 ± 3.29	7.00 ± 2.68	1.24	0.038*
e’-L (cm/s, $\bar{\text{x}}$ ± s)	11.35 ± 3.11	9.32 ± 2.72	0.02	0.021*
E/e’ ( $\bar{\text{x}}$ ± s)	9.03 ± 3.04	8.98 ± 2.26	0.51	0.951
TVR (m/s, $\bar{\text{x}}$ ± s)	2.44 ± 0.41	2.35 ± 0.40	0.02	0.459
TAPSE (mm, $\bar{\text{x}}$ ± s)	22.72 ± 3.97	14.72 ± 2.50	7.92	<0.001***
IVC (%, $\bar{\text{x}}$ ± s)	0.19 ± 0.12	0.12 ± 0.12	0.18	0.053
LAVI (ml/m^2^, $\bar{\text{x}}$ ± s)	17.79 ± 5.98	17.04 ± 4.78	0.69	0.634
S (cm/s, $\bar{\text{x}}$ ± s)	16.85 ± 2.75	10.92 ± 3.44	0.98	<0.001***
Myocardial injury markers				
cTnI (ng/mL, IQR)	0.00 (0-0.22)	0.30 (0.00,0.50)	1.99	0.04*
NT-proBNP (pg/mL, IQR)	1640.00 (873.80,3870.90)	4745.00 (1255.00,12750.00)	1.70	0.08

SIC group, Sepsis-induced cardiomyopathy group; SEPSIS group, SEPSIS group; LVEF, Left ventricular ejection fraction; TVR, Tricuspid regurgitation velocity; LAVI, Left atrial volume index; e’-S, Interventricular septum e’ velocity; e’-L, Lateral wall e’ velocity; E, early diastolic mitral flow velocity; A, late diastolic mitral flow velocity; E/e’, E/e’ ratio; E/A, E/A ratio; TAPSE, tricuspid annular systolic excursion; S, systolic tricuspid annular velocity; IVC-CI, inferior vena cava collapse index; GLS, global longitudinal strain; VTI, time-integrated left ventricular blood flow velocity; cTnI, cardiac troponin I; nt-proBNP, N-terminal pro-B-type natriuretic peptide; *P < 0.05, **P < 0.01, ***P < 0.001.

### Gut microbiome diversity in patients with sepsis-induced cardiomyopathy

The sequencing dilution curves for all the samples were flat (**Supplementary Figure 1**), indicating that the current sequencing depth was sufficient to cover the vast majority of the microbial species in the samples and that the data were suitable for subsequent analysis. We assessed the internal structure of the microbial communities and intergroup differences via both α diversity and β diversity.

**Figure 1:**
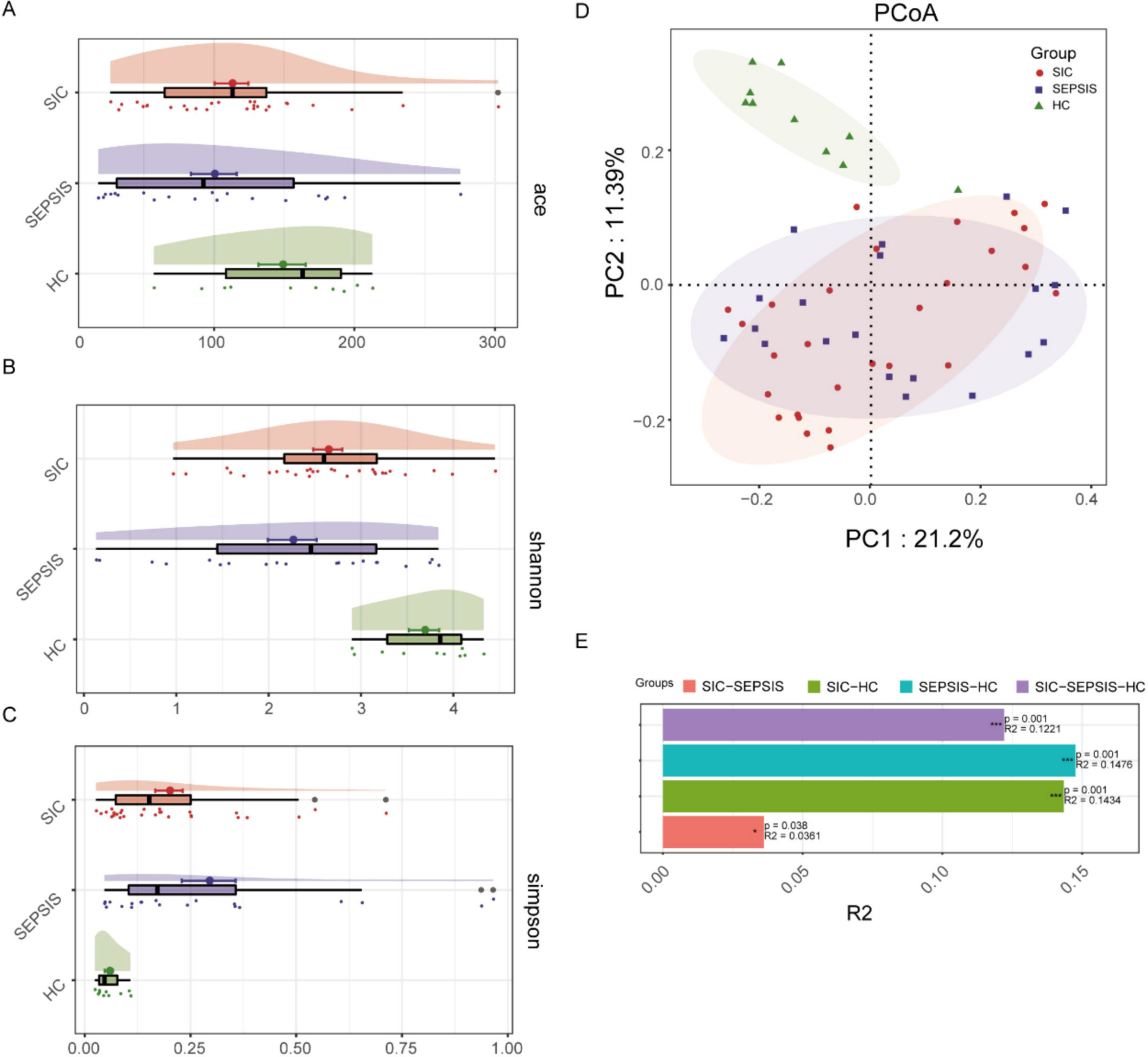
Comparison of gut microbiota diversity among the three groups. **(A–C)** α-diversity was assessed using the ACE index, Shannon index, and Simpson index, with P values of 0.072, 0.001, and 0.001, respectively. **(D)** β-diversity was evaluated by principal coordinate analysis (PCoA) based on unweighted UniFrac distances. Each point represents an individual sample: red indicates the sepsis-induced cardiomyopathy group (SIC), purple indicates the sepsis group (SEPSIS), and green indicates the healthy control group (HC). **(E)** Permutational multivariate analysis of variance (PERMANOVA) revealed a significant difference in the overall microbial community structure (R²=0.122, P = 0.001). *P<0.05, **P<0.01, ***P<0.001.

In terms of α diversity, the Shannon and Simpson indices were significantly different among the three groups (P<0.01). The Shannon index in the SIC and SEPSIS groups was significantly lower than that in the HC group (P<0.05), the Simpson index in the SIC and SEPSIS groups was significantly greater than that in the HC group (P<0.05), and the ACE and Chao indices were significantly different between the SEPSIS and HC groups (P = 0.040, P = 0.03). (**Figures 1A–C**; **Supplementary Figure 2**; **Supplementary Table 1**).

In the β diversity analysis, principal coordinate analysis (PCoA) based on unweighted UniFrac distances revealed that the sample points of the SIC and SEPSIS groups were completely separated from those of the HC group. The sample points of the SIC and SEPSIS groups both overlapped and were partially separated, indicating that the gut microbiota of the SIC and SEPSIS groups differed significantly from that of the HC group overall (P<0.05), whereas the SIC and SEPSIS groups exhibited relatively smaller differences in their gut microbiota overall. PERMANOVA confirmed significant differences in the gut community structure among the three groups (R²=0.122, P = 0.001). Although the differences in the gut microbiota between the SIC and SEPSIS groups were not as pronounced as those between the SIC and HC groups were, significant differences still existed overall (P = 0.038, **Figures 1D, E**).

### Changes in the gut microbiota structure in patients with S sepsis-induced cardiomyopathy

**Figure 2:**
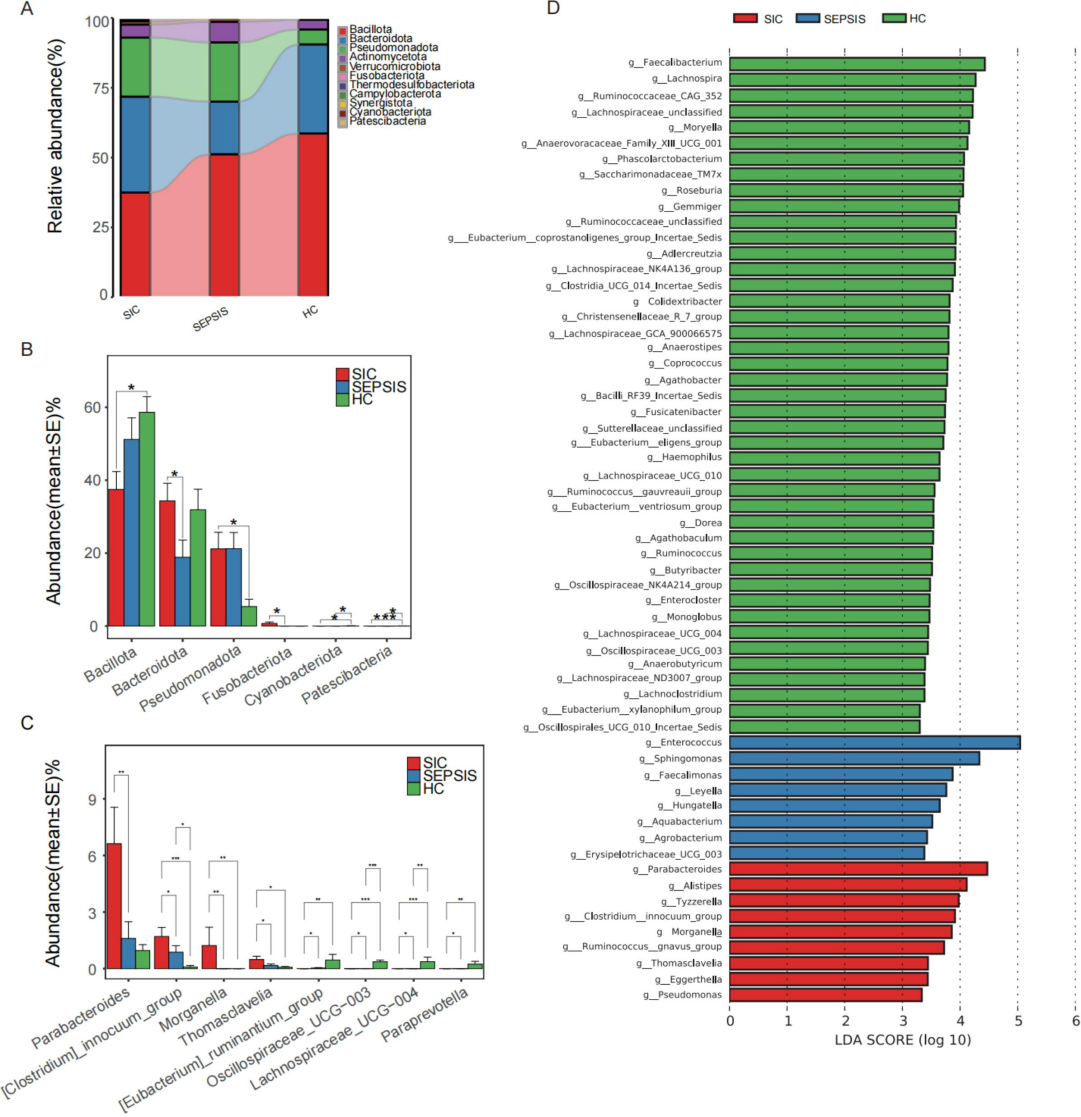
Analysis of gut microbiota distribution and taxonomic differences among the three groups. **(A)** Stacked bar chart at the phylum level, showing the relative abundances of the 11 predominant phyla identified across the study subjects. **(B)** Kruskal-Wallis (K-W) test revealed significant alterations in the abundance of 6 phyla among the three groups. **(C)** K-W test indicated significant differences in 8 key bacterial genera identified across the groups. **(D)** Linear discriminant analysis effect size (LEfSe) was employed to identify key differential taxa at the genus level among the groups, displaying only those with a linear discriminant analysis (LDA) score threshold>3. *P<0.05, **P<0.01, ***P<0.001.

Compared with the SEPSIS group, the SIC group presented a downward trend in the relative abundances of *Bacillota* and *Actinomycetota* at the phylum level, while the relative abundance of *Bacteroidota* was significantly greater (0.34 vs. 0.18, P = 0.0198), and the *Bacillota/Bacteroidota* ratio was significantly lower (P<0.05). Compared with the HC group, the SIC group presented significant decreases in the relative abundances of *Bacillota* and *Pseudomonadota* (0.37 vs 0.58, P = 0.01; 0.21 vs 0.05, P = 0.023), whereas the relative abundance of *Actinomycetota* tended to increase, and the *Bacillota/Bacteroidota* ratio tended to decrease (**Figures 2A, B**).

At the genus level, the relative abundances of 71 bacterial genera differed significantly among the three groups (all P<0.05) (**Supplementary Figure 3**). We selected bacterial genera that presented significant differences and consistent trends between the SIC group and the other two groups for further analysis. Among these genera, the abundances of genera such as *Clostridium innocuum* group, *Morganella*, and *Thomasclavelia* were significantly elevated in the SIC group, whereas the relative abundances of *Oscillospiraceae_UCG-003, Lachnospiraceae_UCG-004, Paraprevotella*, and *Eubacterium ruminantium* group were significantly reduced in the SIC group (all P<0.05) (**Figure 2C**).

LEfSe analysis results indicated that *Parabacteroides* had the highest LDA value in the SIC group, making it the primary characteristic enriched genus in this group; therefore, this genus was selected for further study. *Enterococcus* was the primary enriched genus in the SEPSIS group, whereas *Faecalibacterium* and other genera were the primary enriched genera in the HC group (**Figure 2D**).

### Changes in serum metabolites in patients with sepsis-induced cardiomyopathy

**Figure 3:**
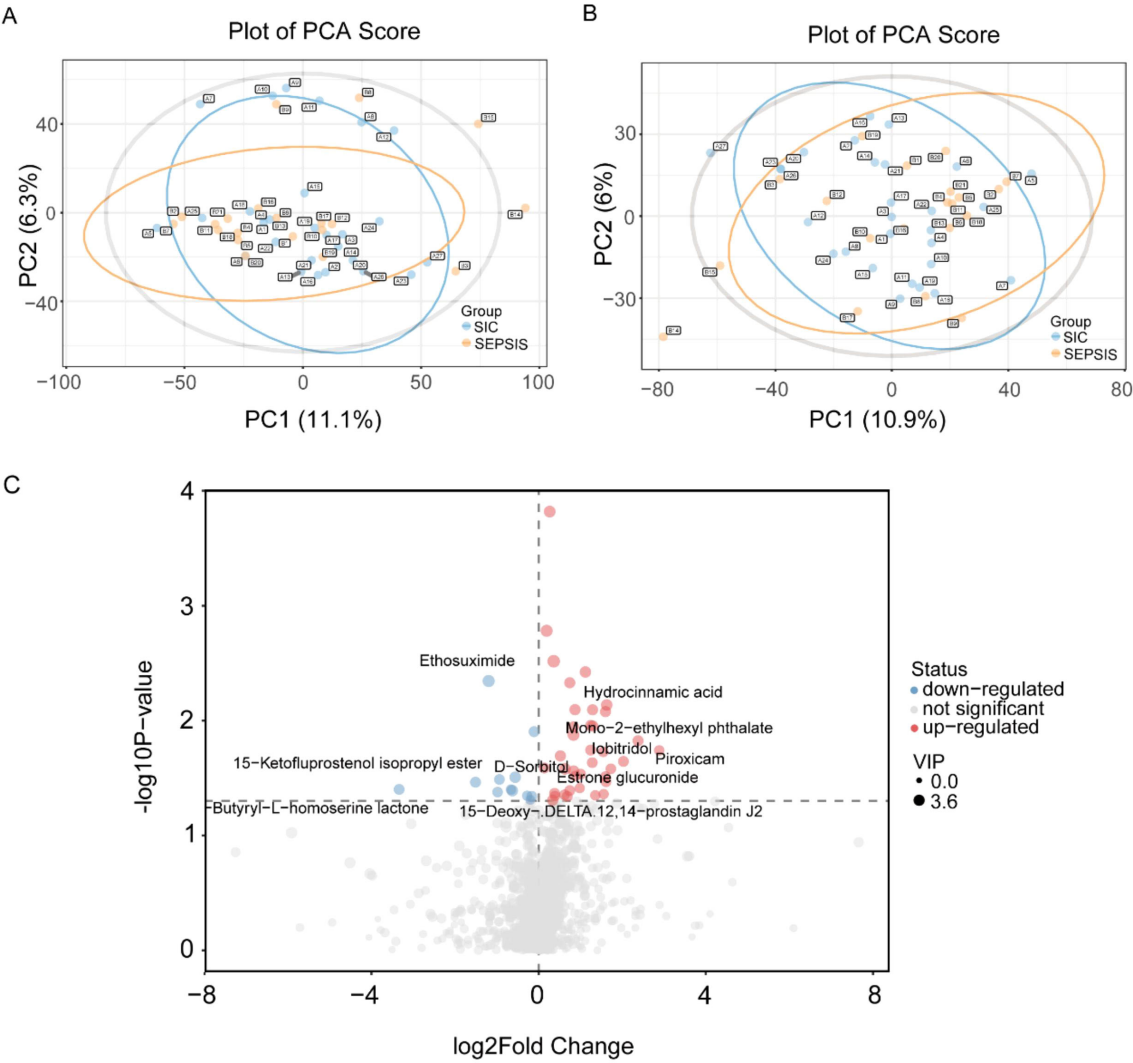
Analysis of differential serum metabolic profiles between patients with sepsis-induced cardiomyopathy and those with sepsis. **(A, B)** Principal component analysis (PCA) plots of the serum metabolic profiles in positive and negative ion modes, respectively. Each point represents an individual sample, and the ellipses indicate the 95% confidence intervals. The sepsis group (SEPSIS) is shown in orange, and the sepsis-induced cardiomyopathy group (SIC) is shown in blue. **(C)** Volcano plot of differential serum metabolites in SIC patients. Blue dots represent down-regulated metabolites, red dots represent up-regulated metabolites, and gray dots represent metabolites that did not meet the criteria for significant differential expression.

A total of 1,303 metabolites were detected in positive and negative ion scanning modes (**Supplementary Table 2**). Principal component analysis (PCA) based on the relative abundances of all the metabolites revealed that the metabolic distributions of the samples from the SIC and SEPSIS groups both partially overlapped and distinctly separated. (**Figures 3A, B**).

**Figure 4:**
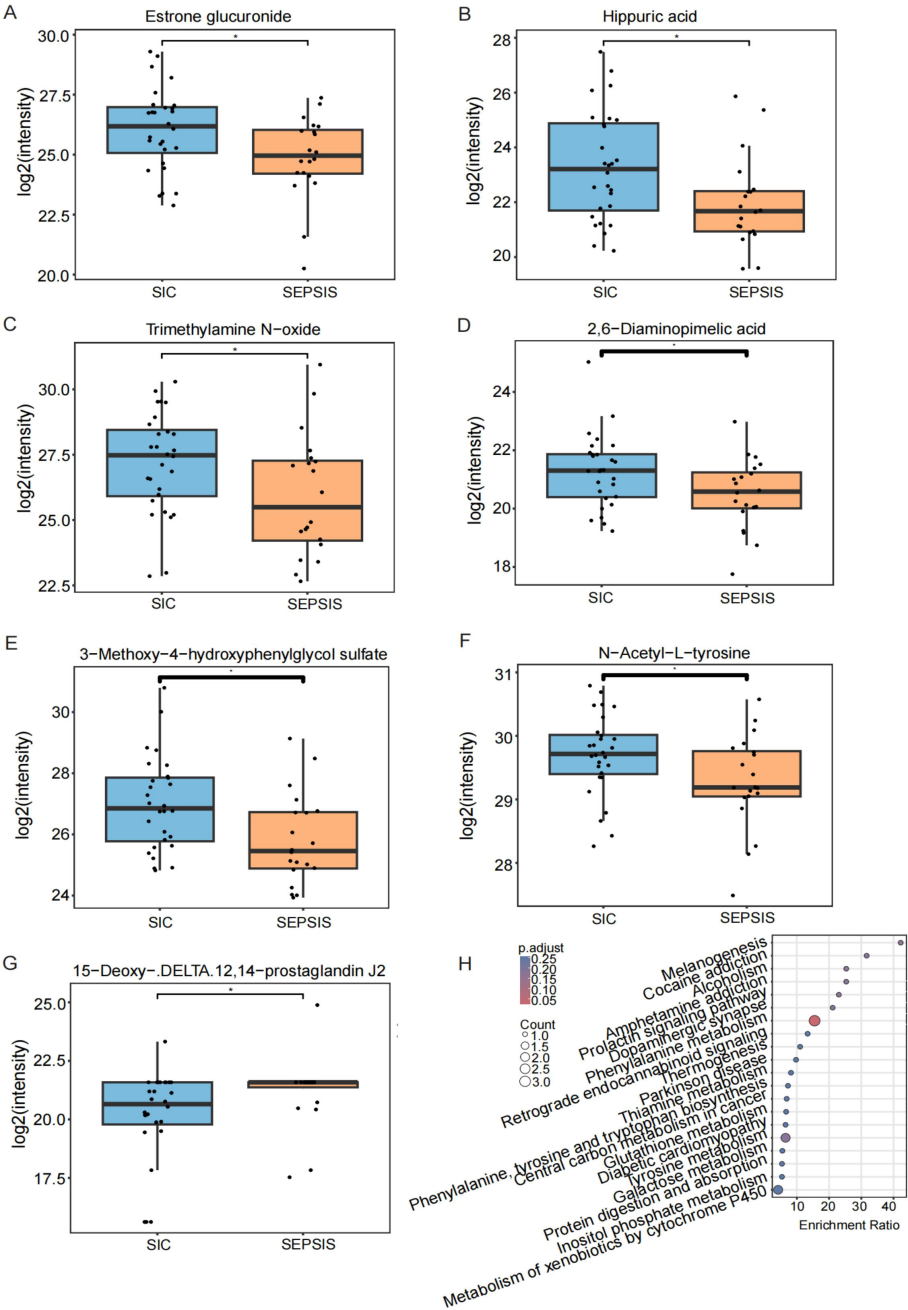
Distribution of key differential metabolites and KEGG pathway enrichment in the SIC group. **(A–G)** The differential distribution of metabolites between the two groups is shown (*P<0.05). **(H)** Bubble chart of KEGG pathway enrichment for the differential metabolites. The color represents the P value, where a shift toward red indicates a smaller P value and greater enrichment significance, and a shift toward blue indicates a larger P value and lower enrichment significance. The size of the bubble corresponds to the number of differential metabolites detected.

Using VIP≥1 and P<0.05 (t test) as screening criteria, a total of 49 differentially expressed metabolites were identified between the two groups. A volcano plot illustrates the differential expression of metabolites between groups, with 37 significantly upregulated and 12 significantly downregulated metabolites in the SIC group (**Figure 3C**; **Supplementary Table 3**). In SIC patients, the serum levels of differentially expressed metabolites such as hippuric acid, estrone glucuronide, trimethylamine N-oxide, 3-methoxy-4-hydroxyphenylglycol sulfate, 2,6-diaminopimelic acid, and N-acetyl-L-tyrosine were significantly elevated (all P<0.05), whereas the serum levels of differentially expressed metabolites such as 15-deoxy-Δ12,14-prostaglandin J2 were significantly decreased (P<0.05) (**Figures 4A–G**).

KEGG pathway enrichment analysis of these significantly differentially expressed metabolites revealed that they primarily participate in functional pathways, including phenylalanine metabolism, tyrosine metabolism, and arachidonic acid metabolism (**Figure 4H**).

### Correlation analysis of the gut microbiota, serum metabolites, and clinical indicators in patients with sepsis-induced cardiomyopathy

**Figure 5:**
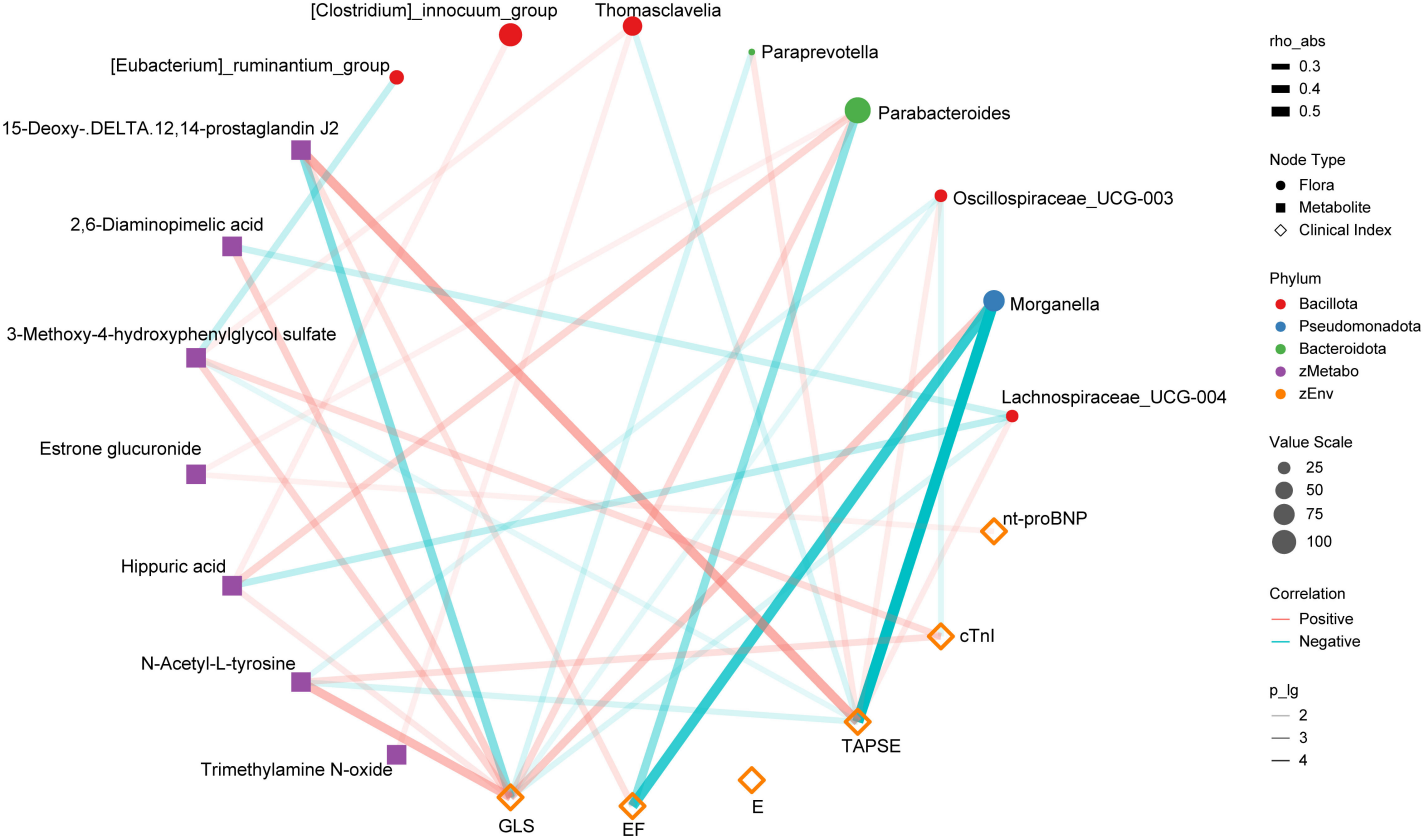
Correlation network analysis of selected differential bacterial genera, differential metabolites, cardiac ultrasound variables, and myocardial injury markers in the SIC group. In the network, connecting lines indicate a significant correlation between two nodes, with red lines denoting positive correlations and blue lines denoting negative correlations. The thickness of a line represents the strength of the correlation, while its transparency corresponds to the negative logarithm of the correlation P-value. In the periphery of the network, solid circles represent gut bacterial genera, with colors indicating their respective phyla. The size of a solid circle reflects the relative abundance of the corresponding genus. Purple squares represent serum differential metabolites. Orange hollow diamonds represent cardiac ultrasound variables and myocardial injury markers.

**Figure 6:**
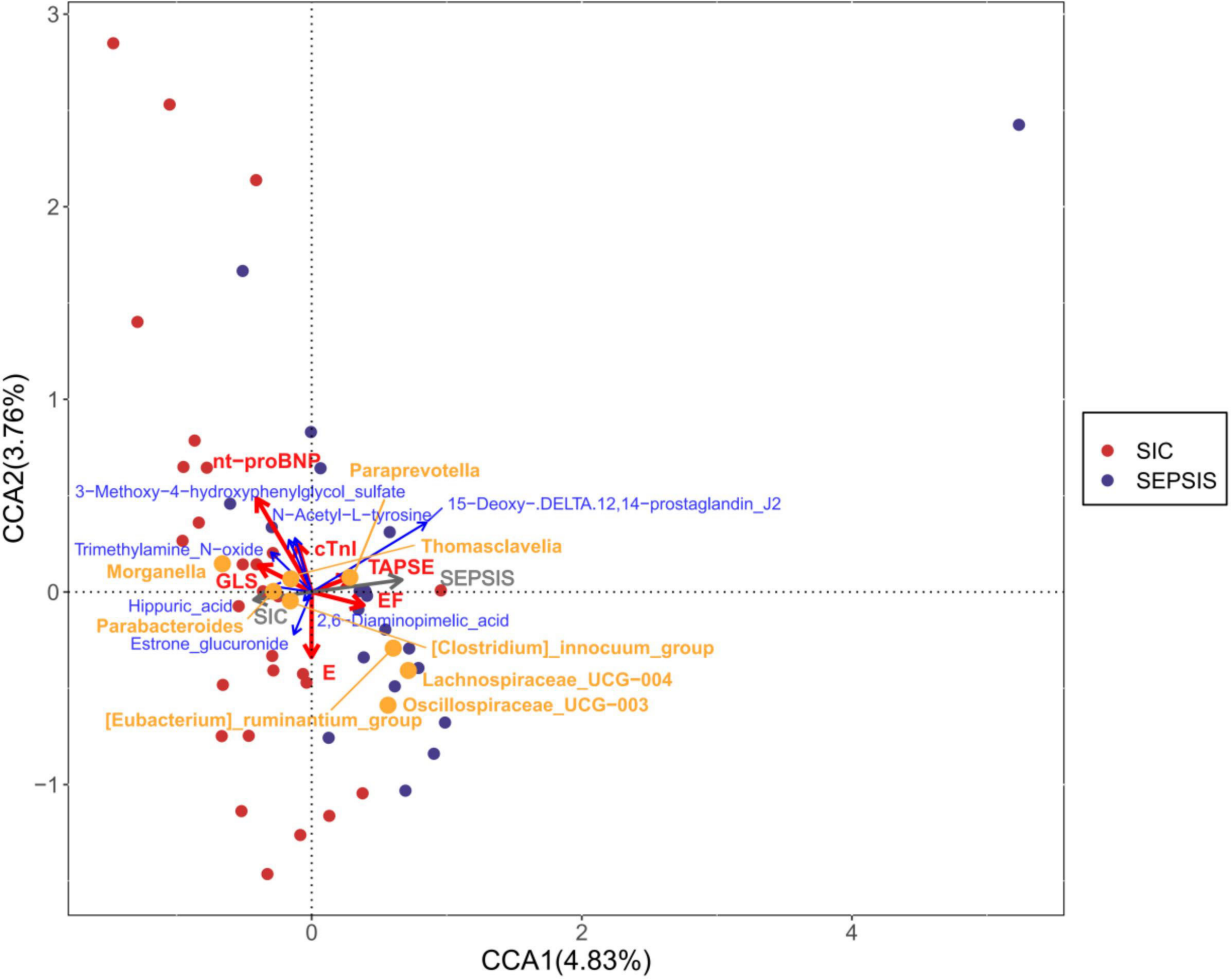
CCA plot of gut microbiota, serum metabolites, and clinical indicators associated with SIC diagnosis in patients from the SIC group and the SEPSIS group. CCA1 and CCA2 represent the first and second principal ordination axes, respectively; the values in parentheses indicate the percentage of microbial variation explained by the corresponding axis. Red dots: samples of the SIC group; blue dots: samples of the SEPSIS group; orange dots: gut bacterial genera. Arrows: the magnitude of contribution of environmental factors to the differentiation of microbial community structure. Gray arrows: the direction of gut microbiota differentiation; blue arrows: serum metabolites; red arrows: clinical indicators associated with SIC diagnosis. A longer arrow indicates a greater contribution. The angle between arrows indicates the direction of correlation: an angle<90° denotes a positive correlation between two factors with consistent variation trends; an angle>90° denotes a negative correlation with opposite variation trends; an angle=90° denotes no significant correlation. CCA, canonical correlation analysis; SIC, sepsis-induced cardiomyopathy group; SEPSIS, SEPSIS group.

Differential bacterial genera and metabolites in SIC patients were strongly and significantly correlated with echocardiographic parameters and myocardial injury biomarkers. (**Supplementary Figure 4**; **Supplementary Tables 4–6**). Among these genera, the 8 key gut bacterial genera significantly differentially expressed in the SIC group presented particularly close associations with key serum metabolites and clinical indicators, leading to the construction of a correlation network diagram. The results revealed significant correlations between the key differentially expressed bacterial genera and key serum metabolites identified in the SIC group and cardiac echocardiographic variables and myocardial injury markers. The relative abundance of the *Parabacteroides* genus was significantly positively correlated with estrone glucuronide and hippuric acid levels (r=0.290, P = 0.045 and r=0.369, P<0.01). Additionally, this genus showed a significant positive correlation with GLS (r=0.358, P = 0.012) and a significant negative correlation with LVEF (r=−0.420, P<0.01); the relative abundance of the *Oscillospiraceae_UCG-003* genus was significantly negatively correlated with N-acetyl-L-tyrosine (r=−0.295, P = 0.038) and was also significantly negatively correlated with GLS and cTnI (r=−0.287, P = 0.047 and r=−0.298, P = 0.039) and was significantly positively correlated with TAPSE (r=0.307, P = 0.033); the relative abundance of the *Lachnospiraceae_UCG-004* genus was significantly negatively correlated with hippuric acid and 2,6-diaminopimelic acid (r=−0.352, P = 0.013 and r=−0.333, P = 0.020), was negatively correlated with GLS (r=−0.304, P = 0.035), and was significantly positively correlated with TAPSE (r=0.309, P = 0.032) (**Figure 5**). To integrate the holistic relationships across multiple omics, we performed CCA. The results revealed that samples from the SEPSIS and SIC groups were clearly divided into two clusters in the ordination space. The metabolite 15-Deoxy-.Δ.12,14-prostaglandin J2 and the clinical indicator NT-proBNP, indicated by the longest arrow lengths, were the primary environmental factors driving the differentiation of the microbial community structure. Genus groups such as *Morganella*, *Parabacteroides*, *Clostridium innocuum* group, and *Thomasclavelia* were positively correlated with the distribution in the SIC group, whereas *Eubacterium ruminantium* group, *Oscillospiraceae_UCG-003*, *Lachnospiraceae_UCG-004*, and *Paraprevotella* were negatively correlated. Furthermore, there was a broad correlation between key bacterial genera, serum differentially abundant metabolites, and clinical indicators related to SIC diagnosis (**Figure 6**).

## Discussion

Our data agree with earlier findings on dysbiosis in SIC, which is consistent with the findings of previous limited studies (Chen et al., 2022; Deng et al., 2023) reporting dysbiosis in SIC patients, thereby providing preliminary support for our results. However, these studies focused primarily on comparisons between SIC patients and non-SIC patients and did not elucidate the characteristics of microbial evolution during the progression from a healthy state to sepsis and subsequently to SIC. Our study analyzed and compared the gut microbiota of SIC patients with that of non-SIC sepsis patients and healthy individuals, revealing that SIC patients exhibit unique gut microbiota characteristics. This is manifested as follows: at the phylum level, during the progression from a healthy state to sepsis and subsequently to SIC, the relative abundance of *Bacillota* gradually decreased; compared with that in patients in the SEPSIS group, the *Bacillota/Bacteroidota* ratio was significantly lower in SIC patients; at the genus level, compared with those in patients in the SEPSIS and HC groups, the relative abundances of opportunistic pathogens such as *Parabacteroides*, *Morganella*, *Clostridium innocuum* group, *Thomasclavelia* and *Paraprevotella* were significantly greater, whereas the relative abundances of probiotic genera such as *Oscillospiraceae_UCG-003* and *Lachnospiraceae_UCG-004* were significantly lower.

The *Bacillota/Bacteroidota* ratio is often regarded as a marker of gut microbiota dysbiosis (Grigor'eva, 2020), suggesting that gut microbiota dysbiosis progresses as the condition evolves from a healthy state to sepsis and ultimately to SIC. The *Parabacteroides* genus plays a crucial role in maintaining intestinal homeostasis and regulating immunity and metabolism; however, under specific conditions, it can also transform into an opportunistic pathogen (Cui et al., 2022; Liu et al., 2025). Studies have shown that *Parabacteroides distasonis* is positively correlated with cholesterol, low-density lipoprotein, and cardiac creatine kinase isoenzymes (Liu et al., 2019), and its relative abundance is elevated in patients with hypertension (Yan et al., 2017), suggesting that it may be a potential pathogen in cardiovascular disease. As opportunistic pathogens, *Clostridium innocuum* group is associated with a high 30-day mortality rate in infected patients and is frequently accompanied by sepsis, shock, and infective endocarditis (Chen et al., 2022; Ioannou et al., 2023). Its pathogenicity may depend on lipid rafts, which may activate inflammatory pathways—including NOD2/NF-κB—thereby exacerbating inflammatory responses and causing damage to distant organs (Wu et al., 2023). Among patients with bloodstream infections caused by the genus *Morganella*, 12.6% developed myocardial infarction, and 25.3% developed congestive heart failure; these cardiovascular complications significantly increased patient mortality (Laupland et al., 2022). The high mortality rate may be related to the unique antibiotic resistance mechanisms and virulence factors of bacteria (Liu et al., 2016). The genera *Lachnospiraceae_UCG-004* and *Oscillospiraceae_UCG-003* are considered gut probiotics, and many of their members are short-chain fatty acid (SCFA) producers (Konikoff and Gophna, 2016; Lin et al., 2024). This study revealed that the abundance of these genera was significantly reduced in SIC patients and that *Oscillospiraceae_UCG-003* and *Lachnospiraceae_UCG-004* may play a role in reducing SCFA production. No short-chain fatty acids were detected in the serum metabolites in this study, which may be related to the reduction in a large number of beneficial bacterial genera and impaired short-chain fatty acid production. SCFAs are important alternative energy substrates for the myocardium; sepsis leads to massive depletion of gut probiotics, resulting in SCFA deficiency, which can exacerbate myocardial energy metabolism disorders and induce cardiac dysfunction (Palm et al., 2022; Hatahet et al., 2023). SCFAs also act as important immunomodulators. Through G protein-coupled receptors such as GPR41, GPR43, and GPR109A, as well as epigenetic regulation via the inhibition of histone deacetylases, they promote the development and functional activation of regulatory T cells and suppress the secretion of proinflammatory cytokines (Macia et al., 2015), suggesting that they may exert a protective effect on myocardial function in sepsis via similar pathways.

This study revealed that echocardiographic parameters were significantly more abnormal in patients with SIC than in patients with sepsis, which is consistent with the characteristics of this disease. Our previous research identified the GLS, E-wave, and TAPSE as echocardiographic predictors of SIC, in addition to LVEF (Yang et al., 2025). Tricuspid annular systolic displacement (TAPSE) is a reliable indicator for assessing right ventricular systolic function, and a TAPSE value <16 mm is the standard for diagnosing right ventricular dysfunction (Humbert et al., 2023). Global longitudinal strain (GLS) is a specific indicator for assessing left ventricular systolic function; it is normally negative, and a larger negative value indicates better left ventricular systolic function (Nyberg et al., 2023). This study revealed that in SIC patients, the relative abundances of the intestinal genera *Lachnospiraceae_UCG-004*, *Oscillospiraceae_UCG-003* and *Paraprevotella* were negatively correlated with GLS and positively correlated with TAPSE, indicating that a significant reduction in the levels of these genera is associated with impaired left and right ventricular systolic function. The relative abundances of genera such as *Parabacteroides* and *Morganella* were positively correlated with GLS, whereas those of genera such as *Thomasclavelia* and *Morganella* were negatively correlated with TAPSE, suggesting that significantly elevated levels of these genera are associated with reduced left and right ventricular systolic function. cTnI and nt-proBNP are the most widely used biomarkers for the clinical assessment of SIC; they play complementary and critical roles in identifying myocardial injury, evaluating cardiac function, and predicting prognosis (Ehrman et al., 2018; Pei and Liu, 2023). *Oscillospiraceae_UCG-003* was significantly negatively correlated with cTnI, suggesting that a significant reduction in this probiotic genus may affect myocardial function. These findings indicate a broad correlation between gut microbiota dysbiosis and myocardial injury in SIC patients.

SIC is a metabolic disease (Salami et al., 2024), and its gut microbiota dysbiosis may affect cardiac function through serum metabolites. In the context of sepsis, damage to the intestinal barrier and dysbiosis trigger drastic changes in circulating metabolites, leading to transient, severe fluctuations in metabolite concentrations. This exposes the myocardium to acute inflammation and energy stress, inducing the reversible cardiac dysfunction characteristic of SIC (Li and Zhang, 2026). Microbial metabolites exert a bidirectional effect on cardiomyocytes: on the one hand, short-chain fatty acids and certain indole derivatives have anti-inflammatory and endothelial-protective effects; on the other hand, compounds such as trimethylamine oxide disrupt myocardial energy metabolism (Muttiah and Hanafiah, 2025). Metabolomic studies have confirmed that sepsis is accompanied by widespread metabolic disturbances, which can modulate organ dysfunction (Pandey, 2024). Consistent with these advances in metabolomics research, the nontargeted serum metabolomics analysis in this study further revealed characteristic metabolic disturbances in patients with SIC. Among these, changes in several key differentially expressed metabolites may have significant clinical implications. Hippuric acid is a cometabolite produced by the host and gut microbiota. The results of the present study revealed that the abundance of this genus increased with increasing relative abundances of the genera *Clostridium innocuum* group and *Parabacteroides*. Additionally, hippuric acid was significantly positively correlated with GLS, an indicator of left ventricular systolic function, suggesting an adverse effect on cardiac contractility. In a sepsis model induced by *Escherichia coli* infection, HA enhances M1-like polarization of macrophages and amplifies MyD88-dependent Toll-like receptor ligand-mediated inflammatory responses; moreover, elevated HA levels *in vivo* are significantly associated with mortality in sepsis patients (Ticinesi et al., 2023). Studies have shown that in patients with chronic kidney disease (CKD), high HA levels are associated with atherosclerosis and left ventricular hypertrophy (Mirji et al., 2026); the mechanism may involve the induction of ROS production by mitochondria, thereby exacerbating the atherosclerotic process (Huang et al., 2018). Gut bacteria produce β-glucuronidase (GUS), which converts estrone glucuronide into active estrone, one of the active forms of estrogen, which is believed to have a protective effect on the cardiovascular system (Cotton et al., 2023). This study revealed that as the relative abundance of the *Parabacteroides* genus increased, the serum levels of estrone glucuronide increased, and this increase was significantly positively correlated with the cardiac injury marker NT-proBNP, suggesting that myocardial damage may occur with increasing levels of this metabolite. The mechanism may be related to the decrease in free estrogen levels caused by elevated estrone glucuronide levels. Trimethylamine (TMA) is a purely microbial metabolite whose production depends directly on the metabolism of the gut microbiota; TMA is converted in the liver to trimethylamine N-oxide (TMAO) (Garcia-Fernandez et al., 2025), and its serum levels increase with increasing abundance of *Thomasclavelia*. Trimethylamine N-oxide is a key factor that promotes the onset and progression of cardiovascular disease and is also a risk factor for cardiovascular disease (Spasova et al., 2024). Studies have shown that TMAO can activate proinflammatory signaling pathways such as the NF-κB and MAPK pathways in vascular endothelial cells and smooth muscle cells, impairing vascular endothelial function, inducing vascular inflammation, and exacerbating cardiac dysfunction (Zhou et al., 2025). 15-Deoxy-Δ12,14-prostaglandin J2 is a metabolite of the arachidonic acid epoxidase pathway (Chaffer et al., 2006). Its serum levels are significantly positively correlated with LVEF and TAPSE and negatively correlated with GLS, suggesting that reduced serum levels may be associated with impaired left and right ventricular systolic function. Studies have confirmed that a decrease in the plasma level of postpercutaneous coronary intervention (PCI) is positively correlated with the extent of myocardial injury and that it reduces myocardial cell damage by inhibiting the excessive activation of Ca^2+^/calmodulin-dependent kinase II (CaMKII) (Hu et al., 2025).

To explore the relationships among SIC-associated gut microbiota dysbiosis, serum metabolic changes, and their associations with disease, we performed a Spearman correlation analysis, which revealed a statistically significant correlation among the three factors. To further integrate the evolution of the gut microbiota structure and holistic interrelationships across multiple omics, we performed a CCA analysis (Raykov, 2025). The results revealed that the gut bacterial genera in the SEPSIS and SIC groups were clearly divided into two distinct clusters in the ordination space, suggesting significant differences in the gut microbial community structure between the two groups. Furthermore, gut microbiota dysbiosis, serum metabolic alterations, and clinical indicators in SIC patients exhibit multiple interrelated patterns. These findings, together with the results of the Spearman correlation analysis, confirm from different perspectives that SIC patients exhibit a characteristic gut microbiota–metabolism–cardiac function association pattern distinct from that of sepsis patients, suggesting an interaction between gut microbiota dysbiosis and altered serum metabolism with SIC. The above results further indicate that there is a broad association among characteristic gut dysbiosis, differential serum metabolites, and cardiac dysfunction in SIC patients.

This study has certain limitations: the study sample size was relatively small, and it was a single-center study. The metabolites identified were not subjected to FDR multiple correction. The interactions and associations between the differentially expressed gut bacterial genera and metabolites in SIC and the clinical indicators related to the diagnosis of SIC reflect only statistical correlations. Larger-scale, multicenter studies and animal experiments may be needed in the future to further validate the biological significance of the “gut microbiota–serum metabolites–clinical indicators” association in SIC.

## Conclusion

This study describes the evolution of gut microbiota phylum/genus-level characteristics during the progression from healthy individuals to sepsis and subsequently to SIC, as well as changes in the serum metabolome of SIC patients. Key bacterial genera, differentially expressed metabolites, and clinical indicators associated with SIC diagnosis were all found to be correlated, suggesting that the gut microbiota may interact with SIC either directly or by regulating serum metabolites. These findings provide a theoretical basis for targeting improvements in the gut microbiome to enhance cardiac function and prognosis in SIC patients.

## Data Availability

The data presented in this study have been deposited in the NCBI Data Repository under accession numbers PRJNA1477312 (SIC group and SEPSIS group) and PRJNA691455 (healthy control group).
