# Supplementary material for: Gut microbiota and serum metabolic profiles in patients with sepsis-induced cardiomyopathy and their association with the disease
Source: Front Cell Infect Microbiol. 2026 Jul 10;16:1811654. doi: 10.3389/fcimb.2026.1811654 (PMC13395620; doi:10.3389/fcimb.2026.1811654)
Supplement: Supplementary file 7 [file Table7.docx]

| 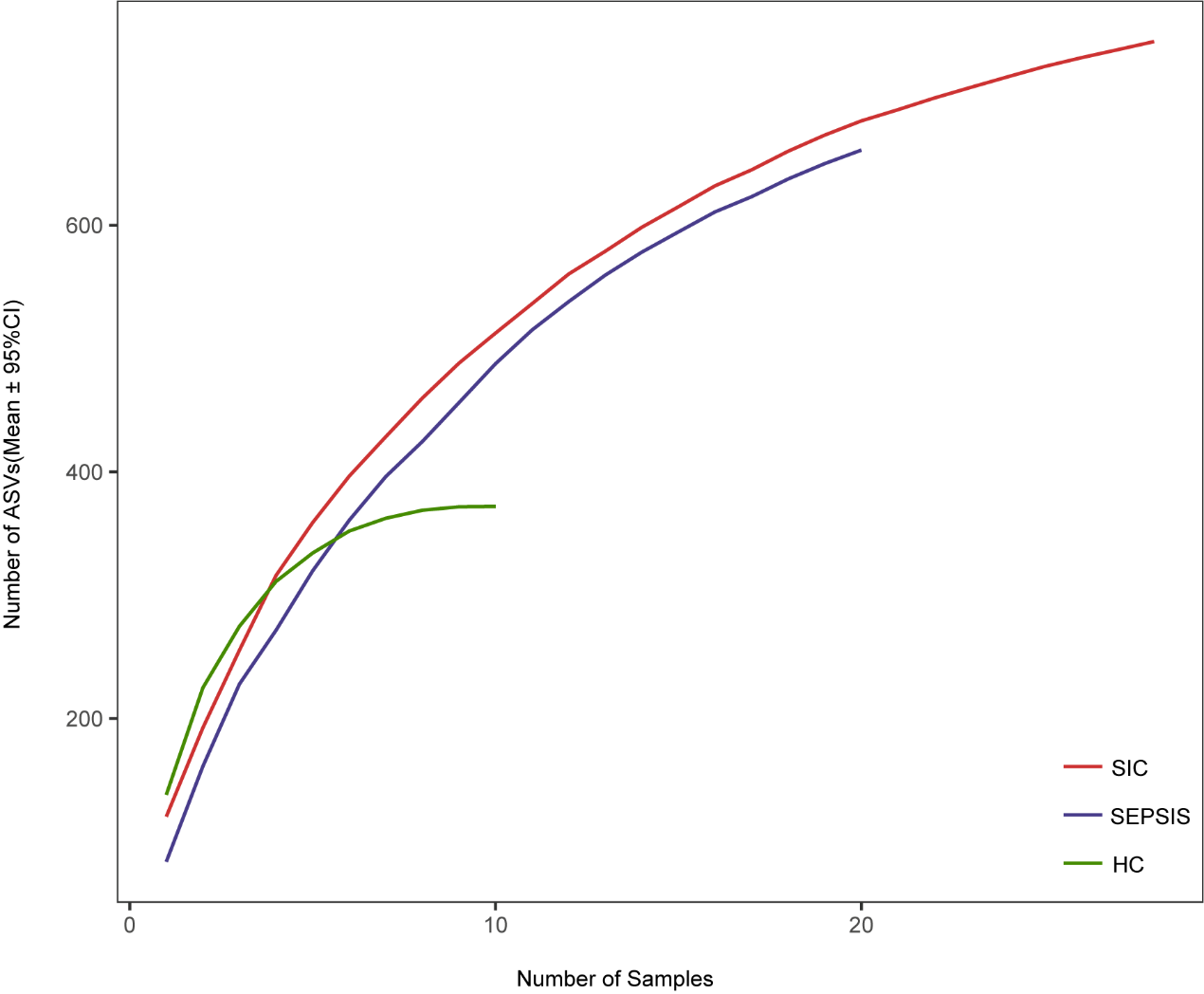 |
| --- |
| Supplementary Figure 1: Sparsity curves. The curves for each group are nearly flat, indicating that the sequencing data is sufficient. Red represents the sepsis-induced cardiomyopathy (SIC) group, purple represents the sepsis (SEPSIS) group, and green represents the healthy control (HC) group. |

| 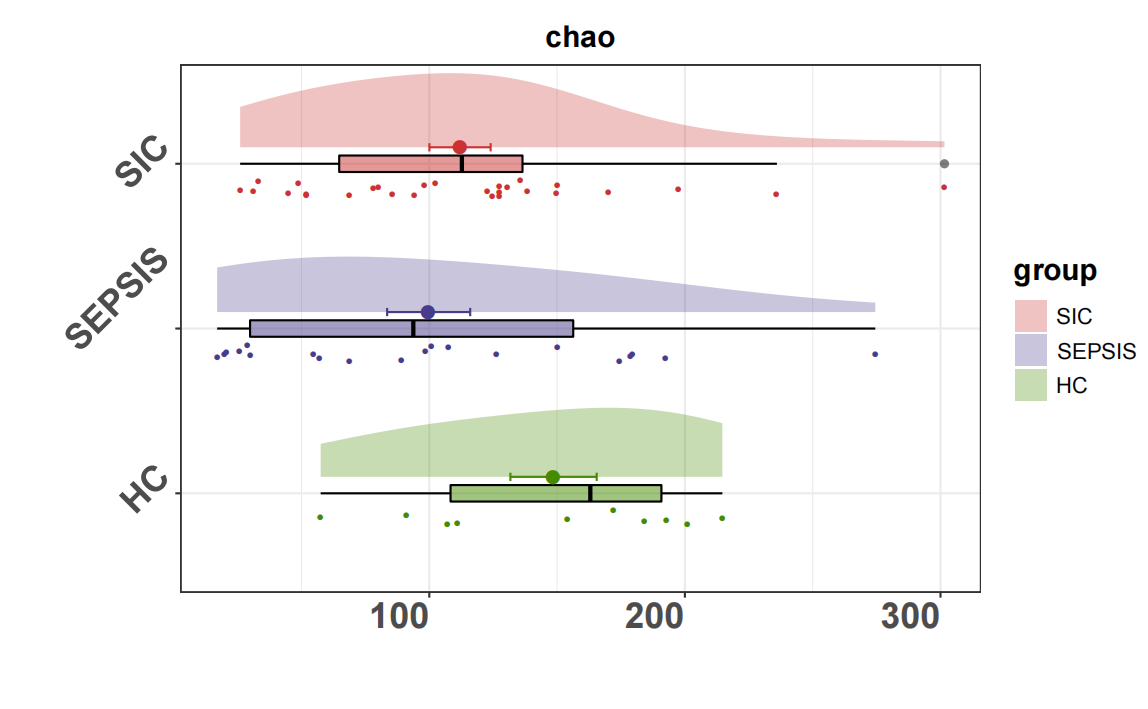 |
| --- |
| Supplementary Figure 2: Chao index of gut microbiota across the three groups.SIC: Sepsis-induced cardiomyopathy group SEPSIS: SEPSIS group HC：healthy control group |

| 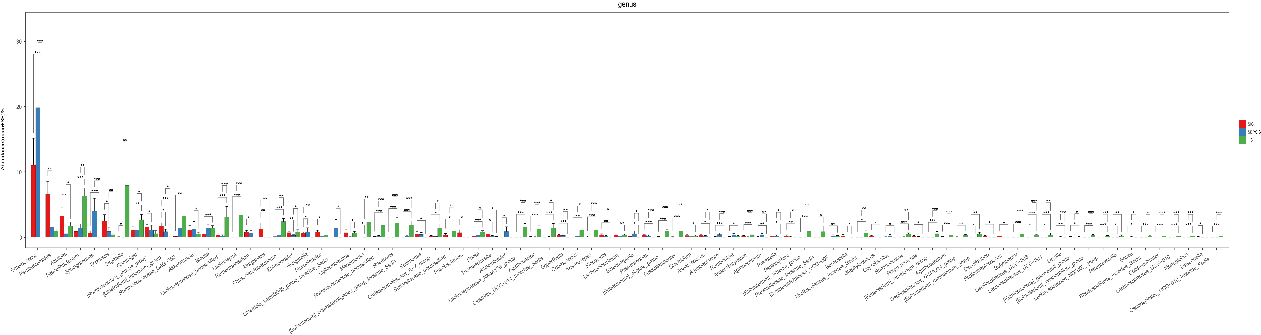 |
| --- |
| Supplementary Figure 3 There were significant differences in the relative abundance of gut bacterial genera among the three groups. Red represents the sepsis-induced cardiomyopathy group (SIC), purple represents the sepsis group (SEPSIS), and green represents the healthy control group (HC). *P < 0.05, **P < 0.01, ***P < 0.001. |

| 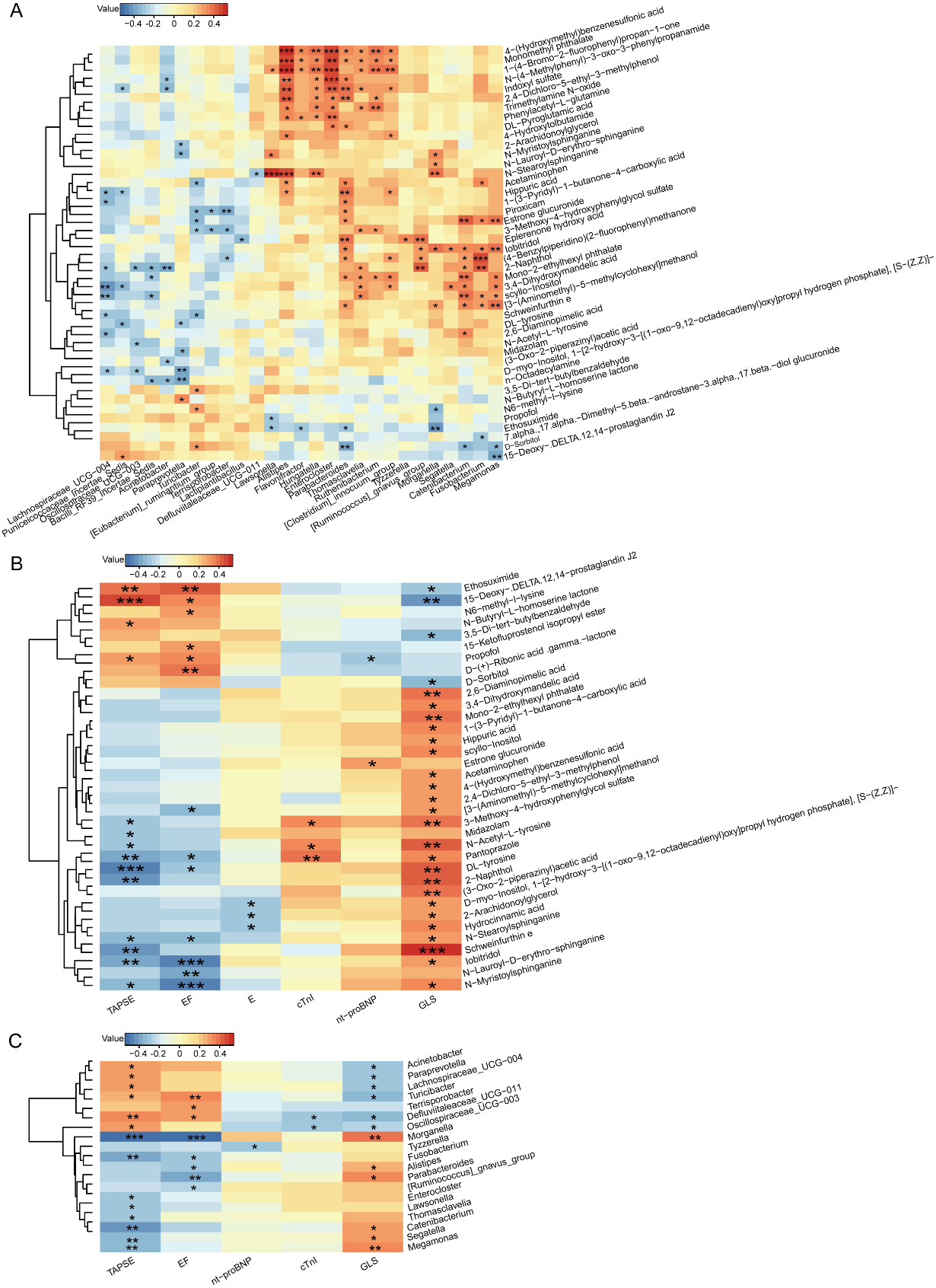 |
| --- |
| Supplementary Figure 4 Analysis of correlations between gut microbiota, serum metabolites, cardiac echocardiographic variables, and markers of myocardial injury in patients with sepsis-associated cardiomyopathy and sepsis. (A) Heatmap showing correlations between differentially expressed gut bacterial genera and serum metabolites. Columns represent differentially expressed gut bacterial genera, and rows represent serum metabolites. (B) Heatmap showing correlations between serum differentially expressed metabolites and cardiac echocardiographic variables and myocardial injury markers. Columns represent cardiac echocardiographic variables and myocardial injury markers; rows represent serum differentially expressed metabolites. (C) Heatmap showing correlations between differentially expressed gut bacterial genera and cardiac echocardiographic variables and myocardial injury markers. Columns represent cardiac echocardiographic variables and myocardial injury markers; rows represent differentially expressed gut bacterial genera. Color blocks indicate the strength of correlation: red represents a positive correlation, blue represents a negative correlation, and darker colors indicate a stronger correlation. *P < 0.05, **P < 0.01, ***P < 0.001. |
