# Supplementary material for: Gut microbiota and serum metabolic profiles in patients with sepsis-induced cardiomyopathy and their association with the disease
Source: Front Cell Infect Microbiol. 2026 Jul 10;16:1811654. doi: 10.3389/fcimb.2026.1811654 (PMC13395620; doi:10.3389/fcimb.2026.1811654)
Supplement: Supplementary file 8 [file Table8.docx]

Supplementary Table 1: Comparison of Clinical Treatment-Related Variables Between the Two Groups

| **Variable** | **SEPSIS (n=20)** | **SIC (n=28)** | **F/χ^2^/t** | **P** |
| --- | --- | --- | --- | --- |
| Vasoactive Drugs |  |  |  |  |
| Epinephrine(ug/kg/min,IQR) | 0.00(0.00,0.00) | 0.00(0.00,0.00) | -1.74 | 0.08 |
| Norepinephrine(ug/kg/min, IQR) | 0.30(0.06,0.50) | 0.45(0.20,0.80) | -1.89 | 0.06 |
| Dopamine(ug/kg/min,IQR) | 0.00(0.00,0.00) | 0.00(0.00,0.00) | -1.49 | 0.13 |
| Dobutamine(ug/kg/min, IQR) | 0.00(0.00,0.00) | 0.00(0.00,0.00) | -1.40 | 0.16 |
| Endotracheal intubationn(%) | 17（85%） | 24（86%） | 0.01 | 1.00 |
| Antibiotic Use |  |  |  |  |
| Carbapenems n(%) | 13（65%） | 18（64.28%） | -0.07 | 0.95 |
| Cephalosporins n(%) | 3（15%） | 5（17.85%） | 0.03 | 1.00 |
| Glycopeptides n(%) | 3（15%） | 5（17.85%） | 0.03 | 1.00 |
| Penicillins n(%) | 0（0%） | 1（3.57%） | 0.12 | 1.00 |
| β-lactamase inhibitor combination preparations n(%) | 10（50%） | 12（42.85%） | -0.07 | 0.77 |
| Oxazolidinones n(%) | 4（20%） | 6（21.42%） | 0.01 | 0.90 |
| Nitroimidazoles n(%) | 0（0%） | 2（7.14%） | 0.17 | 0.50 |
| Aminoglycosides n(%) | 2（10%） | 1（3.57%） | -0.13 | 0.36 |
| Tetracyclinesn(%) | 3（15%） | 5（17.85%） | 0.03 | 1.00 |
| Quinolones n(%) | 2（10%） | 2（7.14%） | -0.05 | 1.00 |

Note: SIC: Sepsis-associated cardiomyopathy group; SEPSIS: SEPSIS group
